# Temporal and Spatial Analysis Techniques as Potential Tools for Combating the HIV Epidemic among Young Brazilian Amazonian People: An Ecological Study

**DOI:** 10.3390/tropicalmed7070137

**Published:** 2022-07-16

**Authors:** Andrey Oeiras Pedroso, Dulce Gomes, Sara Melissa Lago Sousa, Glenda Roberta Oliveira Naiff Ferreira, Aline Maria Pereira Cruz Ramos, Sandra Helena Isse Polaro, Laura Maria Vidal Nogueira, Eliã Pinheiro Botelho

**Affiliations:** 1 Programa de Pós-Graduação em Enfermagem, Federal University of Pará, Belém 66075-110, Brazil; andrey.pedroso@ics.ufpa.br (A.O.P.); sara.sousa@ics.ufpa.br (S.M.L.S.); glendaf@ufpa.br (G.R.O.N.F.); nurse.alinecruz@gmail.com (A.M.P.C.R.); shpolaro@ufpa.br (S.H.I.P.); 2 Departamento de Matemática, Colégio Luís António Verney, University of Évora, 7000-671 Évora, Portugal; dmog@uevora.pt; 3 Departamento de Enfermagem Comunitária, State University of Pará, Belém 66050-540, Brazil; lauramavidal@gmail.com

**Keywords:** HIV, spatial analysis, spatiotemporal analysis, social determinants of health, adolescent young adult

## Abstract

Background: The main goal of this study was to analyze the HIV epidemic temporally and spatially among young people living in Pará, Brazil, from 2007 to 2018. Methods: For the temporal analysis, we employed an integrated autoregression of moving averages model associated with the seasonal trend using the LOESS decomposition method, which allowed for predictions to be made. In the spatial analysis, the techniques of autocorrelation, spatial and spatio-temporal risk analysis, and geographically weighted regression were used. Results: During the study period, there were 8143 notifications of HIV/AIDS cases. The temporal prediction indicated a trend of growth in the incidence rate in the 20–24-year-old group from January 2019 to December 2022 and a trend of stability in the 15- to 19-year-old and 25- to 29-year-old groups. There was a territorial expansion of the HIV epidemic in Pará. Novo Progresso and the Metropolitan Region of Belém (RMB) were the zones with the highest spatial and spatio-temporal risk for HIV. Social determinants including the Basic Education Development Index, the number of physicians per 10,000 inhabitants, and the municipal high school abandonment rate in the municipalities were associated with the risk of HIV/AIDS among young people in Pará. Conclusions: To eliminate HIV among young people in Pará, the access to treatment, diagnosis, and preventive healthcare services should be expanded. Sexual and reproductive health education should be reinforced in schools and communities. Furthermore, it is necessary to promote social equity and fight HIV stigma.

## 1. Introduction

Globally, it is estimated that 3,400,000 million [95% confidence interval = 2,000,000–4,900,000] young people live with human immunodeficiency virus (HIV), which causes acquired human immunodeficiency syndrome (AIDS). Although the number of new infections in young people (15–24 years) has decreased by 46% between 2010 and 2019 [1], the problem persists. Of every seven new HIV infections in 2019, two were in young people. While in some regions the rate of HIV detection among young people has decreased (i.e., east and south of the African continent), in others it has increased (i.e., Eastern Europe, Central Asia, Middle East, North Africa, and Latin America). Such disparity shows that great efforts are required to eliminate HIV among young people by 2030, as proposed by the United Nations (UN) through its 90-90-90 goal [1,2].

In Brazil, every person aged 15 to 29 years is considered young [3]. In this age group, from 2010 to 2020, the detection rate for new HIV infections increased 29% in those aged 15 to 19 years (4.1–5.3) and 20.2% among people aged 20–24 years (23.2–27.9). Although there was a decrease of 4.84% among people aged 25 to 29 years (45.4–43.2), this group had the highest detection rate of all age groups [4]. In Brazil, the northern region had the highest percentage increase (60.62%) in the HIV/AIDS incidence rate between 2009 and 2019 among young people (Figure S1).

Many behavioural factors are associated with the risk of HIV infection among young people, such as the earlier initiation of sexual life, the inconsistent use of condoms, multiple sexual partnerships, the use of smartphone applications for geosocial networks, and behaviour-modifying substances [5,6,7]. However, attributing blame only to these factors is a mistake because the behaviours are shaped by the social determinants of health (SDH). SDH are socioeconomic, cultural, psychological, and/or behavioural factors that influence the occurrence of health problems and their risk factors in the population of a given territory [5,8]. In studying the HIV epidemic, it is necessary to understand the impact of SDH on the specific geography/territory to implement focused and efficient health actions [5]. Spatial and temporal analysis techniques are useful in this task. Spatial analysis techniques allow us to visualize the territories with the greatest epidemiological pressures and correlate the specific problem with territorial SDH. A temporal analysis allows us to assess the impact of public policies aimed at combating the disease [9,10,11].

A mapping of the literature identified only six studies that used purely spatial and/or spatiotemporal analysis techniques to study the HIV epidemic in young people [7,12,13,14,15,16]. None of these studies applied geographically weighted regression (GWR), an analytical technique used to identify the SDH influencing the HIV epidemic. There are also no temporal studies with forecasting applied. In addition, the temporal studies were focused only on the annual variation of the incidence rates, neglecting the important components of a time series such as seasonality and breakpoint changes [12,14,15]. While the breakpoint (or inflection point) analysis shows the exact time point when a time series changes behavior, the seasonality analysis shows the periodicity of the time series fluctuations. These two components are essential for evaluating public policies and to identify the sociopolitical and economic factors associated with the studied phenomenon.

Thus, the objective of this study is to analyse the HIV epidemic temporally and spatially among young people in the state of Pará, Brazil. Additionally, considering that in Brazil the age groups of 15–19, 20–24, and 25–29 years have different behavioural patterns related to the detection rate of HIV/AIDS, we decided to analyse each age group individually.

## 2. Materials and Methods

### 2.1. Study Settings

This is an ecological study in which secondary data from cases of HIV/AIDS reported to the Notifiable Diseases Information System (Sistema de Informação de Agravos de Notificação (SINAN)) between 2007 and 2018 and released by the Public Health Secretariat of the State of Pará were used. The choice of the period 2007 to 2018 aimed at a broad evaluation of possible changes in the pattern of the HIV/AIDS epidemic among the young population after the implementation of the public policies ‘Treatment for All People as Prevention’ (TasP) and the start of compulsory notification of HIV infection, both in 2014 [17,18].

**Figure 1:**
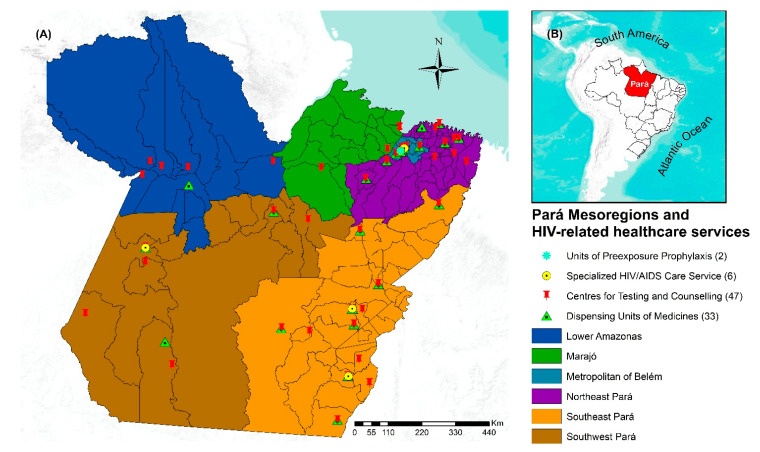
Geographic location of the state of Pará divided into six political mesoregions and HIV-related healthcare services. (**A**) Pará State divided into six political mesoregions and HIV-related healthcare services; (**B**) spatial localization of Pará in Brazil, 2022. Legend: ArcGIS software, version 10.5. Redlands, CA, USA: Environmental Systems Research Institute, Inc.

The study area was the territory of the state of Pará, the second-largest Brazilian state by land area, specifically, an area of 1,247,689.515 km^2^, divided into 144 municipalities, 6 mesoregions (Figure 1), and with a projected population of 8,602,865 in 2019, of whom 31.5% live in rural areas [19]. Additionally, Pará has the third-largest youth population in Brazil, comprising 35.5% of its population in 2020 [17,18]. Pará has the third-lowest human development index (HDI) in Brazil (0.646) and high-income distribution inequality (Gini coefficient: 0.6260) [20]. The state is characterized by densely forested areas with a low population density and a rainy climate, with its highest concentration of health services being in urban areas, which hinders the access of the rural population due to its accessibility only by boat and the long travel times [21].

Regarding policies to combat HIV, Pará has insufficient resources, with only 33 Dispensing Units of Medicines located in 28 municipalities [22], six units of Specialized HIV/AIDS Care Services, 47 centers for testing and counselling, and two units of preexposure prophylaxis in the capital [23]. In 2019, Pará and its capital, Belém, were the fourth-leading state and third-leading Brazilian capital, respectively, with respect to the highest HIV detection rates (2019 Epidemiological Bulletin).

### 2.2. Study Data Set

The study population consisted of all cases of HIV/AIDS in young people from Pará reported by SINAN between 2007 and 2018. Only the reports of young people aged 15 to 29 years residing in municipalities in Pará were included in the study.

The following variables were collected: diagnosis (HIV or AIDS), date of diagnosis, age, sex, race/colour, education level, municipality of residence, zone, and exposure category. The data were placed in a Microsoft Excel spreadsheet, version 16.0 (Microsoft Corp., Redmond, WA, USA), grouped by municipality, and double-checked. The incidence rate was calculated by dividing the number of reports of HIV/AIDS in young people by the young population living in the respective municipalities and the years. The results were multiplied by 100,000. The total population of each municipality was that estimated by the Brazilian Institute of Geography and Statistics (Instituto Brasileiro de Geografia e Estatística (IBGE)) for 2011–2018 or 2007–2010 from the last Brazilian demographic census.

For the GWR analysis, the variables collected from the website of the Amazon Foundation for Studies and Research (FAPESPA) (17) were used and categorized into the following dimensions: education, social inclusion, labor market, health, and safety (Table S1).

### 2.3. Statistical Methods

#### 2.3.1. Temporal Analysis

In the temporal analysis, we considered the monthly HIV/AIDS incidence rate. For the calculation, the number of monthly HIV/AIDS cases was divided by the number of young people living there in the respective year.

Temporal analysis was performed by employing Seasonal and Trend Decomposition with Loess Forecasting (STLF) method [24,25]. The STLF applies an STL decomposition, models the seasonally adjusted data, and with the information of the decompositions, makes future predictions by considering that the parameters observed in the decomposition are repeated in the forecasts. The greater advantage of this approach is that it can make accurate long-terms predictions when compared to ARIMA.

Temporal analysis was performed with the free software RStudio, version 1.3.1093 (©RStudio, PBC. All Rights reserved, Boston, MA, USA, https://rstudio.com/, accessed on March 2021) using the LOESS forecasting model (STLF) [24,26]. The breakpoints and their confidence intervals in the monthly series were also calculated. In the used software, R, a regression calculates the ideal breakpoints in the times series and the confidence intervals [27].

#### 2.3.2. Spatial Analysis

For the spatial analysis, all the cartographic bases used were obtained from the open-access website of the Brazilian Institute of Geography and Statistics and were georeferenced in the universal Mercator projection, zone 22 S, Datum SIRGAS 2000 (Official Datum of Brazil).

ArcGIS software, version 10.5 (ESRI, Redlands, CA, USA), was used for spatial distribution and autocorrelation. Here, the HIV/AIDS incidence rate in young people for four-year periods were used: 2007–2010, 2011–2014, and 2015–2018. This was done to avoid annual fluctuations in the rates. For the calculation, the number of cases in the quadrennium was divided by the average population of young people in the period. The result was multiplied by 100,000.

The global Moran index (*I*) was calculated for each quadrennium and age group. In this analysis, the queen-type matrix was adopted, with the criteria for defining the neighborhood being the contiguity of the edges and nodes and 999 permutations. Only the *I* values with statistical significance (*p* < 0.05) were considered spatial correlations [28]. Following this, the local indicators of the spatial association (LISA) method were applied to visualize the clusters categorized as high-high or low-low values (HH or LL, respectively) and low-high or high-low values (LH or HL, respectively) [29,30].

For the analysis of spatial and spatiotemporal risk to HIV, a spatial and spatio-temporal scan analysis was used employing the software SaTScan^TM^, version 9.7 (developed by Martin Kulldorff in conjunction with Information Management Services Inc., Ltd., Boston, MA, USA) [31]. To calculate the spatial risk, the following criteria were applied: the clusters could not be geographically overlapped, they had to have a maximum size equal to 50% of the exposed population, and there were 999 replicates. For the spatiotemporal risk analysis, the same criteria were used, except that the maximum temporal size of the cluster was considered 50% of the study period. Any relative risk (RR) ≥ 1 with *p* < 0.05 was considered a risk [31]. The confidence intervals for each RR were calculated in R as described [32].

Finally, GWR was performed in MGWR, version 2.2 (ABOR, Phoenix, AZ, USA). First, a linear regression was performed to select the independent variables (SDH) that were correlated with the dependent variable, the HIV/AIDS incidence rate for the 12 years of the study. Next, only the correlations with *p* < 0.05 were analyzed using the ordinary least squares (OLS) method to estimate the model parameters. The generated models were then evaluated for multicollinearity and only the models with an inflation variance factor (VIF) less than 7.5 were considered. The best explainable model was that with the higher R^2^, adjusted R^2^, and smaller Akaike information criterion value (*p <* 0.05). After discarding the spatial dependency of the residuals of the chosen model, the model was evaluated in the GWR [33]. For the GWR, the bandwidth of the kernel type was chosen based on the lower corrected AIC (AICc), with the lowest AICc being selected. The R^2^, adjusted R^2^, AIC, and AICc values were used to compare the OLS and GWR models [34].

### 2.4. Ethics

This study was conducted in accordance with the Declaration of Helsinki and was approved by the Ethics Committee of the Institute of Health Sciences of the Federal University of Pará under Opinion number: 3,488,663.

## 3. Results

Between 2007 and 2018, 8166 new cases of HIV infection were reported in young people aged 15 to 29 years in Pará, Brazil. A total of 23 notifications were excluded: 11 for having no home address in Pará and 12 for being duplicates. Thus, the study population consisted of 8,143 reported cases.

The study population consisted mainly of the 25–29-year age group (47.97%), men (66.46%), people of brown race (76.95%), people with elementary education (36.26%), and people living in urban areas. (85.20%). The highest number of cases was in the heterosexual exposure category (54.41%) (Table 1).

**Figure 2:**
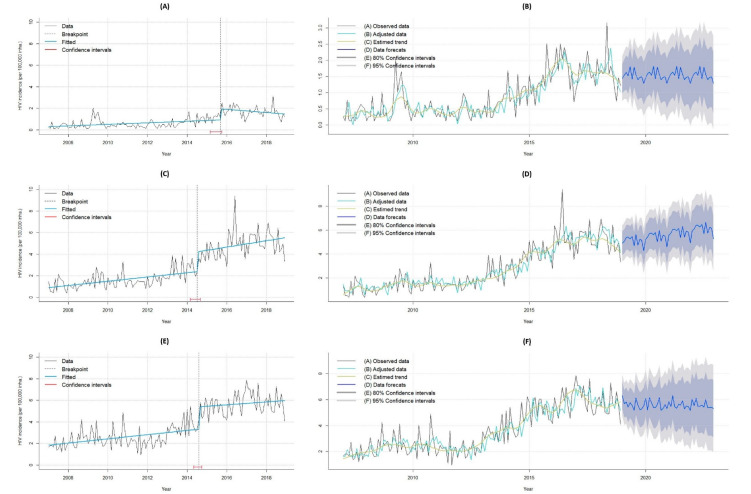
Time series breakpoints of the HIV/AIDS incidence rates in young people in Pará, Brazil (2007–2018), and forecasting projections of the time series from 2019 to 2022, Pará, Brazil. Legend: (**A**,**C**,**E**) show the time series break points of the HIV/AIDS incidence rates in young people in Pará between 2007 and 2018. The black dashed line shows the time of the break point and red trace the confidence interval. (**B**,**D**,**F**) show the forecasting projections of the times series from 2019 to 2022 (blue trace). (**A**–**F**) shows the results for young people aged 15–19, 20–24 and 25–29 years old.

Figure 2A,C,E show the time series of incidence rates among young people aged 15–19, 20–24, and 25–29 years, respectively. The highest incidence rates occurred in young people between 25 and 29 years of age.

The breakpoint of the series of 15- to 19-year-olds occurred in September 2015, with an abrupt increase in the HIV/AIDS incidence rate, followed by a downward trend, while in the other two age groups, the break point in the series occurred in June 2014, with the HIV/AIDS incidence starting to increase, followed by an upward trend.

Table 2 shows the residuals and forecasting parameters of the time series. The projection of the temporal behavior of the HIV/AIDS incidence rate from 2019 to 2022 between the age groups revealed stability in young people aged 15 to 19 (Figure 2B) and 25 to 29 years (Figure 2D), with a chance of error of 45.61% and 19.18%, respectively. In the age group of 20 to 24 years (Figure 2F), there was a growth trend, with a 26.49% chance of error (Table 2).

**Figure 3:**
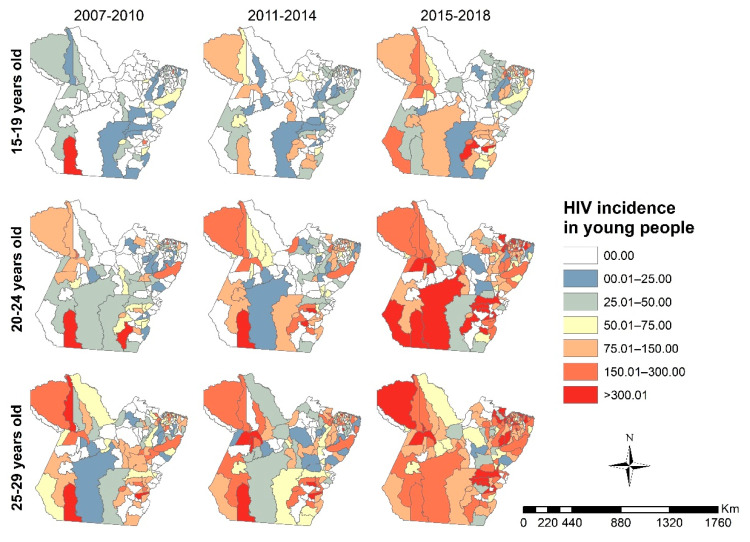
Spatial distribution of the HIV/AIDS incidence rates in young people in Pará for each specific age range (15–19, 20–24, and 25–29 years old) and four-year periods (2007–2010, 2011–2014, and 2015–2018), Pará, Brazil (2007–2018). Legend: ArcGIS software, version 10.5. Redlands, CA, USA: Environmental Systems Research Institute, Inc.

Figure 3 shows the spatial distribution of the HIV/AIDS incidence rate in young people from Pará. The HIV epidemic expanded territorially in the three age groups, but in young people aged 15 to 19 years, the expansion was lesser. The municipalities with the highest incidence rates were in the southwest and southeast Pará mesoregions. There was greater expansion in the northeastern and southeastern regions of Pará.

The global Moran analysis indicated a direct autocorrelation in each age group between 2015 and 2018 (15–19 years: *I* = 0.119800, *p* = 0.01; 20–24 years: *I* = 0.095662, *p* = 0.04; 25–29 years: *I* = 0.158535, *p* < 0.01). The analysis did not show autocorrelation in the quadrennium 2007 to 2010 (15–19 years: *I* = -0.009565, *p* = 0.83; 20–24 years: *I* = -0.013546, *p* = 0.73; 25–29 years: *I* = 0.028288, *p* = 0.28) or 2011 to 2014 (15–19 years: *I* = 0.001494, *p* = 0.86; 20–24 years: *I* = 0.013007, *p* = 0.69; 25–29 years: *I* = 0.074896, *p* = 0.10).

**Figure 4:**
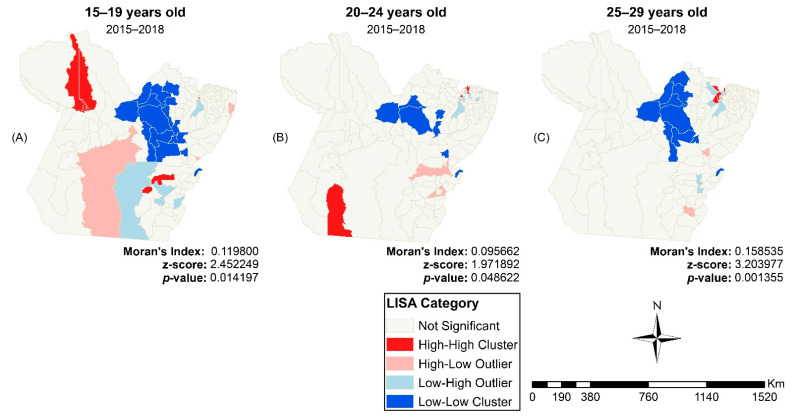
LISA Map of the HIV/AIDS incidence rates between 2015 and 2018 in 15–19 (**A**), 20–24 (**B**) and 25–29 year old young people (**C**) in Pará, Brazil. Legend: ArcGIS software, version 10.5. Redlands, CA, USA: Environmental Systems Research Institute, Inc.

The LISA map revealed low–low incidence clustering in all age groups. The grouping of municipalities with a low–low incidence in people aged 20 to 24 years had the fewest municipalities, namely, Marajó, Lower Amazonas, and Northeastern Pará (Figure 4B). In contrast, the 15–19 group (Figure 4A) and the 25–29 group (Figure 4C) were formed by the municipalities of Marajó, Lower Amazonas, Northeast Pará, Southwest Pará, and Southeast Pará.

**Figure 5:**
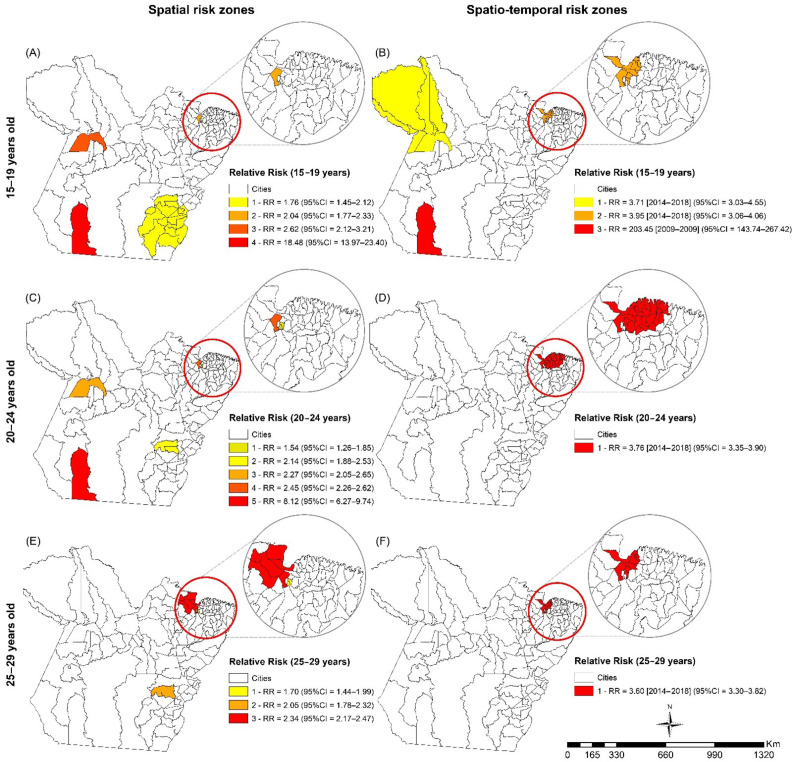
Spatial (**A**,**C**,**E**) and spatio-temporal risk zones (**B**,**D**,**F**) for HIV/AIDS among young people in Pará according to age range group: 15–19 (**A**,**B**), 20–24 (**C**,**D**), and 25–29 years old (**E**,**F**), Pará, Brazil (2007–2018). Legend: CI, confidence interval; RR, relative risk. ArcGIS software, version 10.5. Redlands, CA, USA: Environmental Systems Research Institute, Inc.

Figure 5 shows the spatial and spatiotemporal risk analysis for HIV among young people. In the age group of 15 to 19 years, four spatial risk zones were found, with the highest spatial and spatiotemporal risk observed in the municipality of Novo Progresso (spatial: RR = 18.48, 95% CI = 13.97–23.40, *p* < 0.01; spatiotemporal: RR = 203.45, CI = 143.74–267.42, *p* = <0.001, 2009–2009).

Among the 20- to 24-year-olds, there were five spatial risk zones, with the highest RR in the municipality of Novo Progresso (RR = 8.12, 95% CI = 6.27–9.74, *p* < 0.01). Regarding the spatiotemporal risk, there was only one risk zone, formed by the municipalities of Marajó, the Metropolitan Region of Belém (MRB), and Northeast Pará (RR = 3.76, CI = 3.35–3.90, *p* = <0.001, 2014–2018).

Among the young people aged 25 to 29 years, there were three spatial risk zones, with the highest risk zone encompassing the municipalities of Marajó, RMB, and Northeast Pará (RR = 2.34, 95% CI = 2.17–2.47, *p* < 0.01). There was only one spatiotemporal risk zone, formed by the same three municipalities (RR = 3.60, CI = 3.30–3.82, *p* = <0.01, 2014–2018).

In the GWR, a global regression determined that the best model for the 15- to 19-year-old age group included the Basic Education Development Index (IDEB). For the age groups of 20–24 and 25–29 years, the following variable was added: the number of physicians per 10,000 inhabitants. Additionally, for the age group of 25–29 years, the variable “municipal high school abandonment (MHSA) rate in the municipalities—2018” was added. Table 3 shows that the results obtained through the GWR analysis had better adjustments than the OLS results and that there was no residual autocorrelation.

**Figure 6:**
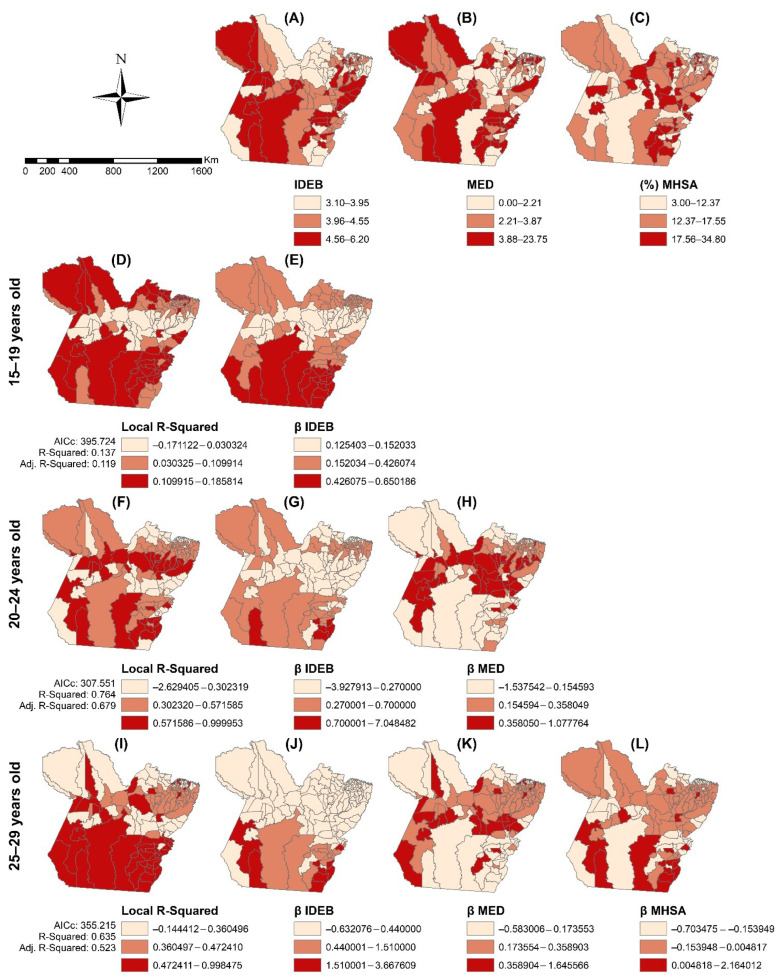
Mapping of the spatial variability of the HIV/AIDS incidence rates promoted by the social determinants of health, Pará, Brazil (2007–2018). Spatial distribution of Basic Education Development Index (IDEB) (**A**); physicians (MED) per 10,000 inhabitants (**B**) and municipal high school abandonment (MHSA) rate (**C**). Coefficients β of IDEB (**E**,**G**,**J**), physicians per 10,000 inhabitants (**H**,**K**) and municipal high school abandonment (MHSA) rate (**L**). (**D**,**F**,**I**) show the local R^2^. Legend: ArcGIS software, version 10.5. Redlands, CA, USA: Environmental Systems Research Institute, Inc.

Figure 6A–C show the spatial distribution of the independent variables, namely IDEB, physicians per 10,000 inhabitants, and MHSA rates, respectively. Figure 6D,F,I show the local coefficients of the determinations of regressions (local R^2^) for young people aged 15–19, 20–24, and 25–29 years old, respectively. Figure 6E,G,J show the β coefficients of the influence of “IDEB” on the HIV/AIDS incidence, indicating a higher risk of HIV in municipalities in the southwest and southeast of Pará associated with high and moderate IDEB, respectively. In municipalities in the northeast and Marajó, the risk of HIV was moderate, and these municipalities were associated with low IDEB. Concerning the “physicians per 10,000 inhabitants”, the risk for HIV was only conferred to those young people aged 20–24 and 25–29 years old (Figure 6H,K), in which the municipalities with a low number of physicians had the higher risk. Figure 6L shows the risk of HIV associated with MHSA rates, with an increased risk of HIV in municipalities in the southeast with high MHSA rates.

## 4. Discussion

### 4.1. Temporal Analyses

The results of this study show the growth of the HIV/AIDS detection rate among young people in Pará, with divergent temporal behaviors between the age groups studied. In young people aged 15 to 19, the HIV/AIDS incidence rate peaked in September 2015, and it was followed by a downward trend, while in people aged 20 to 29 years, the incidence rate started to increase in June 2014 and maintained its upward trend until December 2018.

Such a temporal behavior of the incidence rate among people aged 15 to 19 years might have been due to the HIV testing campaign conducted between 2015 and 2016 in Belém [35], which significantly increased the detection rate of the virus, while due to the discontinuation of this campaign, the virus detection rate decreased again. In the 20–29-year-old group, the increase in HIV/AIDS incidence rate observed in 2014 was due to the compulsory notification of HIV starting that year, as well as the decentralization of tests to the basic health units in 2015.

In Brazil, young people are considered priority populations for the fight against HIV, and in 2007, the Brazilian Ministry of Health launched the Health in School Program in which one of its aspects was the sexual and reproductive health of young people [36]. However, recent studies show a low level of knowledge about the forms of HIV prevention and transmission among Brazilian young students [37].

It is worrying that this study predicts a stable incidence rate of HIV/AIDS among young people aged 15 to 19 (0.01%) and 25 to 29 (0.38%), and an increase among young people aged 20 to 24 years (23.71%) for the period of 2019–2022. Comparing the projections with the data from the Ministry of Health, between 2018 and 2020 there was a 22.22% drop in the number of HIV/AIDS notifications in people from Pará aged 15 to 19 and a 22.02% and 22.07% drop in people aged 20 to 24 and 25 to 29, respectively. However, one must consider the underreporting and low rate of HIV testing resulting from the COVID-19 pandemic [4].

The expansion of testing associated with the immediate initiation of treatment after an HIV diagnosis may promote a decrease in the rate of detection of the virus among young people [38]. In Brazil, in the general public, the expansion of testing and implementation of TasP promoted an immediate decreasing effect on the AIDS detection rate by 62% and on the AIDS mortality rate by 79% [39]. In China, specific counselling for young people was associated with increased testing and it decreased the rate of HIV detection among young people aged 15 to 24 years [40]. However, Pará has still not implemented ART decentralization to the Primary Healthcare network, even as of June 2022. Considering Pará’s large territory and the low socioeconomic conditions of its inhabitants, people experience great difficulty in accessing the 33 UDM belonging to six specialized HIV/AIDS care services. In Manaus, the capital of Amazonas, a state bordering Pará, the decentralization of healthcare for PLWHA to the Primary Healthcare network promoted a greater satisfaction and higher ART adherence among patients [41].

It is not enough to offer sex education in schools if no diagnostic, treatment, and prevention services are available to young people. In London, the continuous and accessible availability of HIV tests, condoms, and antiretroviral therapies promoted a decrease in the rate of HIV detection among homosexuals [42]. The universalization of access to health care is necessary to combat HIV and requires global efforts [43].

Additionally, it is necessary to combat stigma and social prejudice to promote accessibility for all. In the United States, among men who have sex with men, HIV stigma was directly associated with a difficulty in adherence to antiretroviral therapy [44]. Among young Africans, stigma and social prejudice were related to low HIV-testing rates, and self-testing was one strategy indicated as a possible solution [45].

### 4.2. Spatial Analyses

The spatial analysis showed the territorial expansion of HIV/AIDS detection rates among young people living in Pará. The spatial autocorrelation showed LL clusters for all age groups under study in the 2015–2018 quadrennium, which could be due to the low HIV testing rates. In addition to facing historical social inequities, these municipalities have a low coverage of primary healthcare (PHC) and a large proportion of the population have difficulty in accessing health services, largely due to being riparian people living far from urban centers and having the river as their only route of access and locomotion [46].

Novo Progresso was highlighted as having the highest spatial and spatio-temporal (2009) risks for HIV among people aged 15 to 24 and 15 to 19 years old, respectively. Novo Progresso was founded in 1993 and proceeded to experience rapid growth promoted by illegal mining, timber extraction, road construction, livestock, illegal land occupation, and the growth of the mining industry. The high spatio-temporal risk regarding HIV/AIDS noted in 2009 in this municipality can be explained by the increased HIV/AIDS detection in this year. Novo Progresso has reached the peak of its development, with population growth caused by large projects, such as roads and the construction of the Belo Monte Hydroelectric Power Plant in the neighboring municipality, Altamira. This disordered urban development, without investments in infrastructure that support the population’s quality of life, can lead to a favourable environment for sexually transmitted diseases. In 2009, the Edson Royer Institute in Novo Progresso was created to combat violence and prostitution among children and adolescents, pregnancy among adolescent girls, and youth employment in drug trafficking [47,48].

Among people aged 25 to 29 years, the greatest spatial risk for HIV was observed in a cluster made up of the municipalities of Marajó, the RMB, and northeastern Pará. The RMB has the highest population density in the state. This clustering also showed the spatio-temporal risk for HIV among those aged 20 to 29 years between 2014 and 2018. Urbanization facilitates a greater circulation of sexually transmitted infections [49]. In Malawi, it was found that people residing in urban areas have a 2.2 times higher risk of being HIV-positive than their counterparts in rural areas [50]. The fall in spatio-temporal risk between 2014 and 2018 suggests that policies to combat HIV, such as compulsory HIV notification from 2014 and the decentralization of tests in 2015, increased HIV/AIDS diagnoses from 2014 to 2018. Furthermore, this result suggests that the policies fighting HIV are proving to be more efficient in the RMB and northeast region, which can be explained by the fact that these regions have better access to healthcare services and a higher concentration of these services.

The spatial regression showed that the IDEB, number of physicians, and MHSA rates influenced the spatial heterogeneity of the HIV epidemic in Pará. In the southwest municipalities, it was noticed that the risk for an increase in HIV/AIDS incidence was directly influenced by IDEB, while in the municipalities in the southeast this association was indirect. In addition, in southeast municipalities this risk was increased by the low MHSA rate. Although young people with a higher education level are expected to exhibit lower-risk behaviors, studies show that this rule does not always hold [51,52]. Additionally, because southern Pará it is a region with high employment, it is possible that these young people occupy positions with better salaries. Young people with higher incomes with low HIV/AIDS awareness are more likely to be HIV-positive because they have increased exposure to the virus through access to technologies such as geosocial smartphone applications that allow them to engage in more sexual partnerships, sex tourism, and licit and illicit behavior-modifying drugs [6,7].

The risk of HIV was also lower in the municipalities of southern Pará associated with a high number of physicians. Southern Pará has the greatest economic development in the state of Pará, leveraged by the expansion of mining, agribusiness, industries, and large construction projects, such as the construction of a hydroelectric plant. In this region, the urban infrastructure has improved, with primary healthcare services coverage growing by 180% since 2010 and 10 UDM being installed. These facts might have promoted better access to HIV tests, diagnosis, and treatment; consequently, this could have decreased the HIV risk in these municipalities. In Brazil, the Universal Test and Treat (UTT) policy expansion decreased the average AIDS mortality rate by 60% after 2015 [39]. Although no studies have explored how this policy has influenced HIV transmission in Brazil, in sub-Saharan Africa, a three-year population-based study showed that UTT decreased AIDS mortality and HIV transmission by 23% and 32%, respectively [53].

The fact that the municipalities of Marajó and Baixo Amazonas show a moderate risk of HIV directly and indirectly associated with IDEB, respectively, and with MHSA rates raises an alert for the possible increase in the HIV incidence rates in these regions. In addition to having a low concentration of PHC and HIV health-related services, the municipalities of Baixo Amazonas and Marajó are experiencing rapid economic development promoted by agribusiness and livestock and attract immigrants searching for jobs. For example, between 2015 and 2018, the municipalities of Faro (Baixo Amazonas) and Afuá (Marajó) had their gross domestic product increase by 10.12% and 8.82%, respectively [20]. The population growth associated with deforestation and with the lack of investment in urban infrastructure promotes not only the increase in HIV but also other health and social problems [54].

### 4.3. Study Limitation

Since this was an ecological study, we could not correlate the results obtained with causal factors. One must consider underreporting, which was further increased during the COVID-19 pandemic due to the displacement of professionals in the pandemic scenario, compromised information quality, and other restrictions imposed because of the pandemic, such as the interruption of HIV testing in health services; there has been a 41% decrease in the number of HIV tests globally [1,55]. In Pará, the data show a 7.45% reduction in antiretroviral dispensation during the COVID-19 pandemic (2019 until September 2021) [56]. Thus, the epidemiological scenario may be worse than that presented, and future studies need to evaluate the impact of the pandemic [55].

## 5. Conclusions

The results show an expected increase in the HIV/AIDS incidence rate from 2019 to 2022 in people aged 20 to 24 years and a trend towards stability in those aged 15 to 19 and those aged 25 to 29 years. During the study period, there was a territorial expansion of the HIV/AIDS epidemic among the young people in Pará. Novo Progresso and the RMB were the zones with the highest spatial and spatio-temporal risks for HIV. The IDEB, number of physicians per 10,000 inhabitants, and MHSA rates were the SDH influencing the HIV spatial variability in Pará.

To eliminate HIV in Pará, the decentralization of HIV-related healthcare services to the PHC network should be immediately implemented in Pará to achieve better ART adherence and healthcare, and consequently, to decrease HIV spread and AIDS mortality. In addition, sexual and reproductive health education should be reinforced in schools and communities and HIV stigma should be eliminated. Furthermore, mediatic campaigns, policies, and other public strategies to fight HIV should consider regional differences to reach their expected efficiencies.

## Figures and Tables

**Table 1:** Sociodemographic characteristics of young people notified with HIV/AIDS in Pará, Brazil (2007–2018).

Variables	15–19 Years		20–24 Years		25–29 Years		15–29 Years	
	(*n* = 1055)	%	(*n* = 3182)	%	(*n* = 3906)	%	(*n* = 8143)	%
Gender								
Male	595	56.40	2185	68.67	2632	67.38	5412	66.46
Female	459	43.51	996	31.30	1272	32.57	2727	33.49
unanswered	1	0.09	1	0.03	2	0.05	4	0.05
Race/skin color								
White	103	9.76	305	9.59	356	9.11	764	9.38
Black	42	3.98	159	5.00	232	5.94	433	5.32
Yellow	15	1.42	20	0.63	15	0.38	50	0.61
Blown	825	78.20	2440	76.68	3001	76.83	6266	76.95
Native Indian	3	0.28	17	0.53	17	0.44	37	0.45
Unanswered	67	6.35	241	7.57	285	7.30	593	7.28
Schooling								
Elementary	427	40.47	1088	34.19	1438	36.82	2953	36.26
High School	341	32.32	1062	33.37	1143	29.26	2546	31.27
Graduation	39	3.70	325	10.22	421	10.78	785	9.64
Unanswered	248	23.51	707	22.22	904	23.14	1859	22.83
Living zone								
Urban	912	86.45	2742	86.17	3284	84.08	6938	85.20
Rural	88	8.34	272	8.55	384	9.83	744	9.14
Peri-urban	16	1.52	16	0.50	35	0.90	67	0.82
Unanswered	39	3.70	152	4.78	203	5.20	394	4.84
Exposure category								
Homosexual	311	29.48	1028	32.31	971	24.86	2310	28.37
Bisexual	83	7.87	272	8.55	293	7.50	648	7.96
Heterosexual	524	49.67	1612	50.66	2295	58.76	4431	54.41
Blood	-	0.00	7	0.22	10	0.26	17	0.21
Perinatal	31	2.94	52	1.63	57	1.46	140	1.72
Unanswered	106	10.05	211	6.63	280	7.17	597	7.33

**Table 2:** Residuals and forecasting parameters of the temporal models for HIV/AIDS incidence rates time series, Pará, Brazil (2007–2018).

Test	15–19 Years	20–24 Years	25–29 Years
	Test Statistic *p*-Value		
Ljung–Box	0.04	<0.01	0.06
Box–Pierce	0.86	0.91	0.99
Rank test	0.78	0.81	0.78
Turning Point	0.14	0.59	0.02
Lilliefors (Kolmogorov–Smirnov)	<0.01	0.02	<0.01
Shapiro–Wilk normality test	<0.01	<0.01	<0.01
F test	0.04	0.86	0.79
Model	STL + ARIMA(2,1,1)	STL + ARIMA(0.1,1) with drift	STL + ARIMA(2,1,3)
MAPE	45.61464	26.4993	19.18589
MAE	0.6170666	0.560686	0.6170666
RMSE	0.3519083	0.7553249	0.7946913

**Table 3:** Results of the chosen explicative model in the ordinary least squares and in the geographically weighted regressions. Results of the analysis of the AICc, R-squared, adjusted R-Squared, and residuals of the OLS and GWR models used, according to age group, Pará, Brazil (2007–2018). Legend: AICc, corrected Akaike information criterion; OLS, ordinary least squares; GWR, geographically weighted regression.

Analysis	Age Group		
	15–19 Years	20–24 Years	25–29 Years
OLS			
AICc	1521.203	1653.173	1637.290
R^2^	0.071	0.185	0.246
Adjusted R^2^	0.064	0.173	0.230
OLS residues			
Moran’s Index	0.029337	0.053157	0.068410
z-score	1.2472656	1.501993	1.590206
*p*-value	0.21	0.13	0.11
GRW			
AICc	395.724	307.551	355.215
R^2^	0.137	0.764	0.635
Adjusted R^2^	0.119	0.679	0.523
GWR residues			
Moran’s Index	0.023757	−0.006620	0.070442
z-score	1.041916	−0.275607	1.570456
*p*-value	0.29	0.78	0.11

## Data Availability

The datasets analysed during the current study are not publicly available due restrictions apply to the availability of these data. All data in this study were used under license, and so are not publicly available.

## Supplementary Materials

The following supporting information can be downloaded at: https://www.mdpi.com/article/10.3390/tropicalmed7070137/s1, Figure S1: HIV/AIDS detection rate between 2009 and 2019 among young people in Brazil, 2009–2019.; Table S1: Variables used in Geographically Weighted Regression.

## Abbreviations

AIC—Akaike’s information criterion; AICc—corrected Akaike’s information criterion; BIC—Bayesian information criterion; ARIMA—Autoregressive integrated moving average; GWR—Geographically weighted regression; Ndiffs—Number of differentiations; RMB—Metropolitan Region of Belém; SDH—Social Determinants of Health; STL—Seasonal and Trend decomposition using LOESS; STLF—LOESS forecasting model; TB—Tuberculosis.
