# Supplementary material for: Temporal and Spatial Analysis Techniques as Potential Tools for Combating the HIV Epidemic among Young Brazilian Amazonian People: An Ecological Study
Source: Trop Med Infect Dis. 2022 Jul 16;7(7):137. doi: 10.3390/tropicalmed7070137 (PMC9319365; doi:10.3390/tropicalmed7070137)
Supplement: Supplementary file 1 [file tropicalmed-07-00137-s001.zip › tropicalmed-1783164-supplementary.pdf]

Tropical Medicine and Infectious Disease

MDPI

Article

Temporal and Spatial Analysis Techniques as Potential Tools for Combating the HIV Epidemic among Young Brazilian Amazonian People: An Ecological Study

Andrey Oeiras Pedroso [1], Dulce Gomes [2], Sara Melissa Lago Sousa [1], Glenda Roberta Oliveira Naiff Ferreira [1], Aline Maria Pereira Cruz Ramos [1], Sandra Helena Isse Polaro [1], Laura Maria Vidal Nogueira [3] and Eliã Pinheiro Botelho [1,*]

[1] Programa de Pós-Graduação em Enfermagem, Federal University of Pará, Belém 66075-110, Brazil; andrey.pedroso@ics.ufpa.br (A.O.P.); sara.sousa@ics.ufpa.br (S.M.L.S.); glendaf@ufpa.br (G.R.O.N.F.); nurse.alinecruz@gmail.com (A.M.P.C.R.); shpolaro@ufpa.br (S.H.I.P.)

[2] Departamento de Matemática, Colégio Luís António Verney, University of Évora, 7000-671 Évora, Portugal; dmog@uevora.pt

[3] Departamento de Enfermagem Comunitária, State University of Pará, Belém 66050-540, Brazil; lauramavidal@gmail.com

* Correspondence: ebotelho@ufpa.br

Table S1. ariables used in Geographically Weighted Regression.

	**Variable**
	Education
v1	Failure rate in elementary school in the municipalities - 2018
v2	Failure rate in high school in the municipalities - 2018
v3	Dropout Rate in Elementary School in the municipalities - 2018
v4	Dropout Rate in High School in the municipalities - 2018
v5	Basic education development index - IDEB - Public school - for the first five years of school in the municipalities – 2017
v6	Basic education development index - IDEB - Public school - for the 9 years of school in the municipalities – 2017
	**Social inclusion**
v7	Number of Families Assisted in the Bolsa Família Program in the municipalities– 2018
v8	Total families enrolled in the Single Register for Social Programs (CadÚnico) in the municipalities - 2018
v9	Total families enrolled in the Single Register for Social Programs (CadÚnico) with per capita family income of up to 1/2 minimum wage in the municipalities- 2018
	**Labor Market**
v10	Total Employment in Formal Employment in the municipalities – 2018
v11	Average Remuneration (R$) of Formal Worker in the municipalities – 2018
	**Health**
v12	Municipal Family Health Strategy services coverage – 2018
v13	Municipal Basic Healthcare unities coverage - 2018
v14	Municipal Healthcare places coverage per 10,000 inhabitants - 2018
v15	Physicians per 10,000 inhabitants - 2018
v16	Municipal hospital bed coverage per 1,000 inhabitants - 2018
	**Safety**
v17	Youth homicide rate per 100,000 inhabitants – 2018

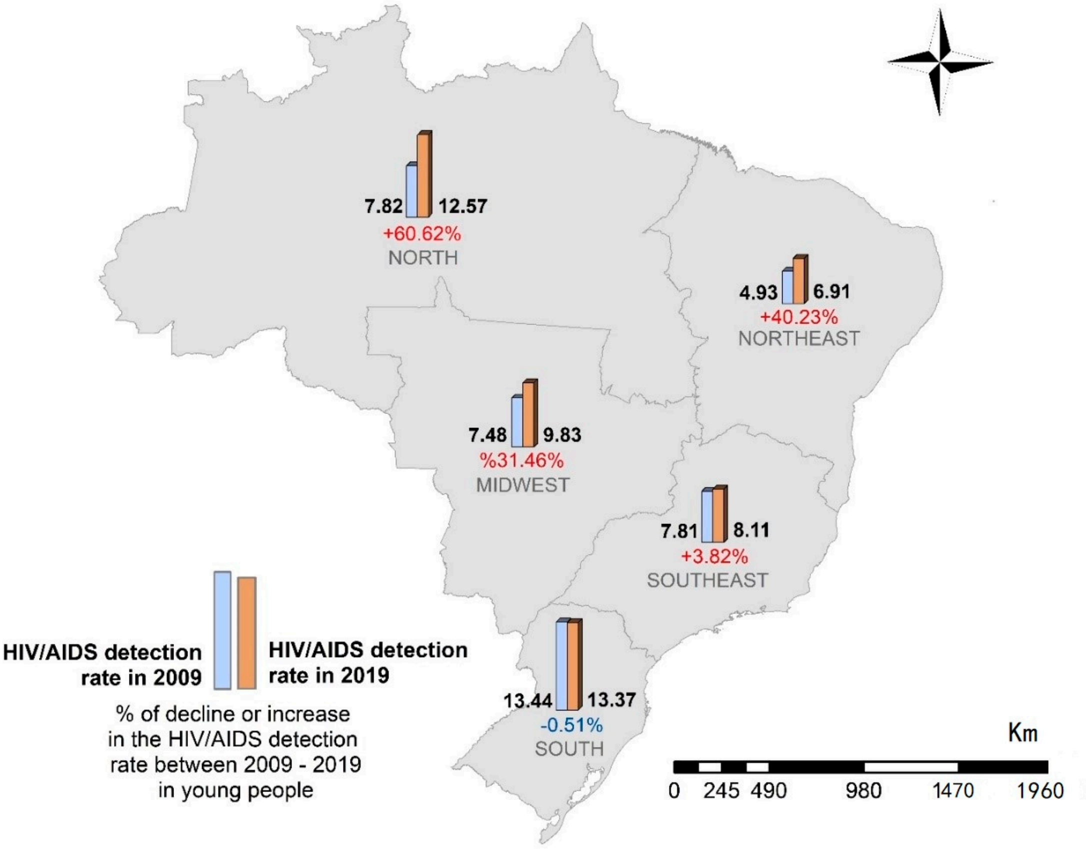

Figure S1. HIV/AIDS detection rate between 2009 and 2019 among young people in Brazil, 2009–2019.
